# Peanut hulls, an underutilized nutritious culinary ingredient: valorizing food waste for global food, health, and farm economies—a narrative review

**DOI:** 10.3389/fnut.2024.1453315

**Published:** 2024-08-19

**Authors:** Suzannah Gerber, Susan B. Roberts

**Affiliations:** ^1^Gerald J. and Dorothy R. Friedman School of Nutrition Science and Policy, Tufts University, Boston, MA, United States; ^2^Geisel School of Medicine, Dartmouth College, Hanover, NH, United States

**Keywords:** food waste, sustainable food systems, peanuts, valorization, food systems approach, plant food, food manufacturing, sustainable technology

## Abstract

Peanut hulls (PHs) are an edible food waste that is an underutilized food source for human consumption. While edible and palatable, currently they are mainly diverted to livestock feed or building materials. Here, we describe existing literature supporting human food valorization of PHs, and propose methods to optimize recapturing nutrients (protein, fiber, phenols and other phytonutrients) lost by treating PHs as waste. Incorporated into common foods, PHs could be processed into functional ingredients to improve nutrient-density with anticipated corresponding positive health outcomes associated with increases in plant foods. Valorization of PHs addresses multiple priorities of the UN Sustainable Development Goals using a Food Systems Approach (FSA) including reducing food waste, increasing economic opportunities for farmers, and increasing the availability of healthy shelf-stable foodstuffs to address food security. Recent advances in sustainable food processing technologies can be utilized to safely incorporate PHs into human food streams. We propose future applications that could make meaningful impacts for food availability and the nutritional composition of common foods like bread and plant-based meat alternatives. While the limited literature on this topic spans several decades, no commercial operations currently exist to process PHs for human consumption, and most literature on the topic precedes the technological “green revolution.” The approaches outlined in this review may help bolster commercialization of this underutilized and nutritious food potentially improving opportunities for multiple global stakeholders.

## Introduction

The global production of peanuts annually is approximately 46.4 million metric tons, of which nearly 22% (>10 million tons) is waste from the hulls (also known as peanut shells) (1). Peanuts are grown and consumed in high volumes on every continent, and except for the U.S. (the fifth largest grower) and China (first) peanut farming is mostly concentrated in lower- and middle-income countries (LMIC) where economic growth opportunities and food security are more scarce, including world growing leaders India, Nigeria, and Sudan, as well as Senegal and Argentina (1).

Reducing food waste (United Nations Sustainable Development Goal (SDG) 12) and increasing the efficiency of human food systems to achieve zero hunger (SDG 2) are global priorities. Valorizing food waste can address both of these as well as SG3, which prioritized health and well-being; SDG 6, which prioritized sustainable water management; SDG 8 which prioritizes economic growth; and, SDG 10, which aims to reduce disparities for those with less access and opportunities (2). Due to the large production and waste volumes of peanuts, upcycling major byproducts like peanut hulls (PHs) would substantially increase the total amount of human food available from current land use and resource inputs which would not only help address hunger, but could potentially improve the cost–benefit and revenue potential for farmers by increasing the sellable volume from existing yields (3, 4). Peanut production utilizes a substantial amount of energy or fuel, fertilizer or other soil amendments, and water, which results in sizable greenhouse gas emissions (GHGs), eutrophication, and other environmental externalities that vary across global growing regions and farming practices, and proportionately increases as farming efficiency decreases (5, 6). In addition to representing inefficient food systems and lost revenue opportunities, food waste itself is a considerable source of GHGs (7, 8). Peanuts are estimated to produce approximately 60,000 MJ/ha^−1^ and of that, over 25% of the potential energy output is in the hulls (6). Considerable labor, power and water is thus spent generating a crop where >22% by weight and >25% of resultant nutrient and energy resources resultant are diverted to low-cost animal feed and other low cost applications that are notably priced even lower than other plant byproducts, in part due to their abundance (9).

Given the high percentage of waste from PHs and the interest to derive greater value from such an abundant resource stream, many approaches to upcycle PHs have been explored. While the most common uses of PHs are animal feed, PHs are also employed as a dry composite material for packaging and industrial fillers, and emerging industries have investigated the potential to use PHs for biofuel and commercial filtration systems (1, 10–12). However, PHs are edible to humans, and thus diverting them to feed, fillers, and filters is a missed opportunity to efficiently manage food systems for human consumption. As food systems struggle to manage the increasing global food demand, more efficient systems will be needed– and recapturing edible side streams are recognized to be an important part of that strategy (13, 14).

**Figure 1:**
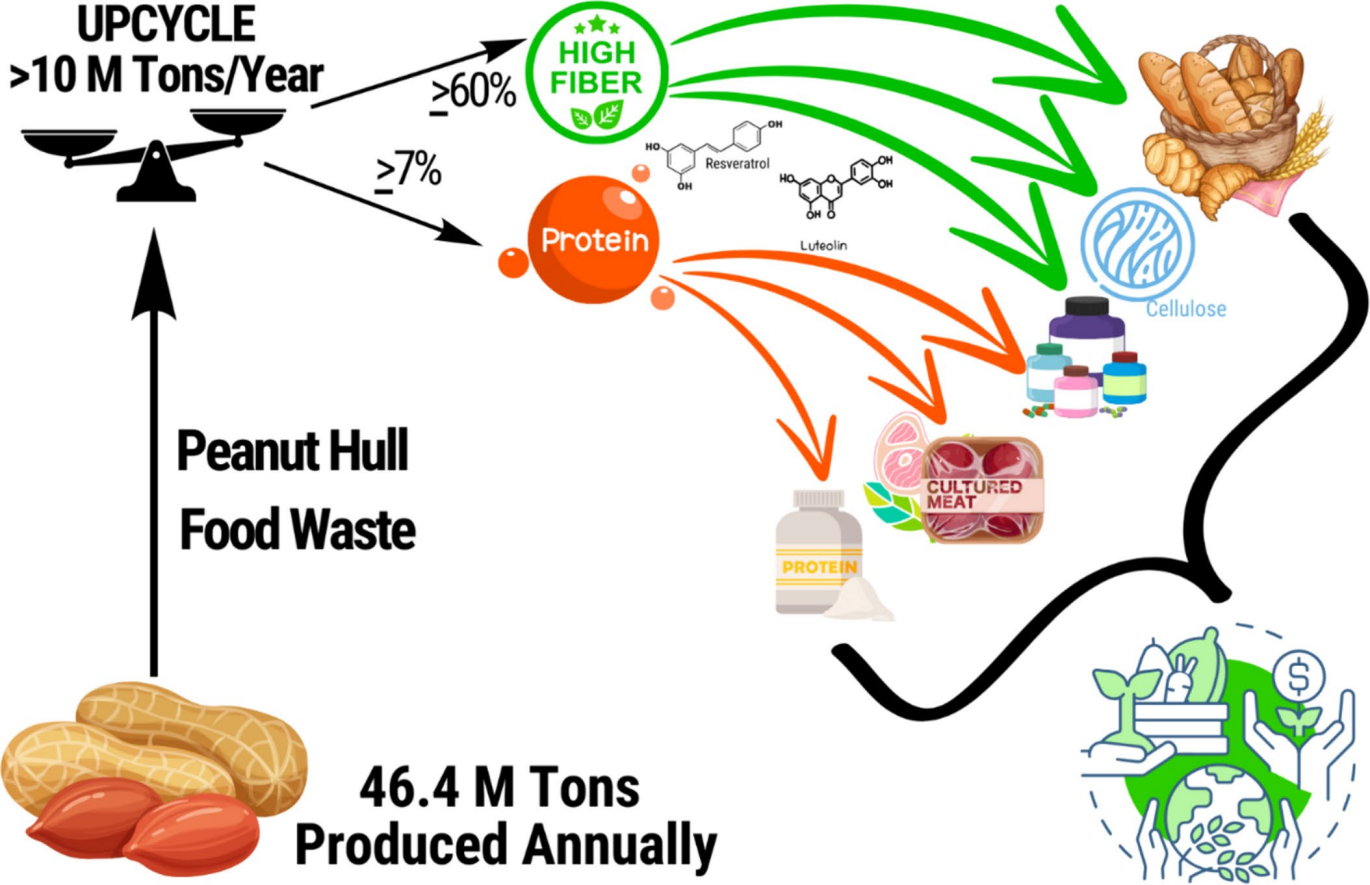
Conceptual model of peanut hull valorization for human food. Approximately 46.4 million tons of peanuts are grown annually around the world, of which 22% is waste from the hulls (>10 million tons). Peanut hull waste results in an annual loss of >6.5 million tons of health-promoting dietary fiber and > 595,000 tons of healthful plant protein (1). Recapturing PHs for human foods has applications that could improve the healthfulness of common foods, such as breads and other baked goods by using it to amend traditional baking flour. PHs also provide an abundant source of functional ingredients, that could join or be used in place of popular ingredients like pea protein for use in foods and nutritional supplements. In this figure peanut hull waste in tons is shown being upcycled in order to recapture major nutrients (protein and fiber) along with beneficial polyphenols (luteolin and resveratrol) for use in high-feasibility examples of bread (and baked goods), cellulose commonly used in food production, in nutritional supplements, in alternative proteins such as plant-based and cultivated meat, and as protein powders for direct consumption. Together, these value-added uses increase the market value of peanuts with significant economic benefits for farmers, represent a more efficient use of food system resources, and reduce food waste and the corresponding environmental impacts. These benefits join the benefits of their specific applications which range from improving nutrient profiles of foods to help offset the burden of cardiometabolic disease, and increasing food and nutrition availability as a means to addressing food and nutrition insecurity.

Human food valorization of PHs offers potentially unique benefits for human nutritional health. Through efficient retention in the human food supply, the overall supply of shelf-stable and nutritious foodstuffs could thereby increase supporting better food and nutrition security. Reclaiming peanut byproducts like hulls for human consumption also presents economic opportunities for farmers by increasing the market value of PHs which could be sold at a premium compared to low-cost animal feed (15). Additionally, by getting more out of every peanut harvest and incentivizing greater byproduct salvage the environmental impacts of peanuts could be reduced relative to the amount of human foodstuffs products, while significantly reducing the impacts of food waste, itself (1, 16–18). Figure 1 depicts the logical framework of this review.

Our aim in this narrative review is to summarize the prior uses and known benefits of reclaiming PHs for human food and to outline approaches to safely process and culinary applications to utilize PHs for human consumption. We focus on how to overcome the common obstacles for the valorization of PHs and present a brief introduction to the benefits, which include increasing the availability of healthy plant foods, reducing food waste and the related environmental impacts, and increasing the sellable tonnage of harvests for peanut farmers.

## Current uses and comparable products

### Medicinal use of peanut hulls

PHs are rich sources of dietary fiber, >60% of the dry weight. By comparison, whole bran cereal is typically 13–25% dietary fiber, and wood pulp—a common source of cellulose-based nutritional fiber supplements—typically has 15–35% fiber by weight (19). PHs are therefore an exceptionally rich source of dietary fiber that can be used to fortify other foods (17). Additionally, protein is approximately 7% of the total weight of PHs, representing nearly 20% calories from protein (20, 21); by contrast, all-purpose wheat-flour has approximately 11% calories from protein. PHs are also a rich source of beneficial phytonutrients from plant foods such as polyphenols, flavonoids, and procyanidins including luteolin and resveratrol which are bioactives commonly used in pharmaceuticals and nutritional supplements and that have been demonstrated to have cardiovascular and anticarcinogenic effects *in vitro* and *in vivo* (1, 20, 22–24). While investigation into the benefits of these phytonutrients is broad, there is some promising literature that discusses the pharmaceutical application of extracts specifically derived from PHs as an adjuvant treatment of cancer (25, 26), as a treatment for hypertension and hyperlipidemia (18, 23, 27), and as an anti-inflammatory for pain management (23, 28). Extracts from PHs of the polyphenol luteolin have also been shown to have anti-diabetic properties in animal models, and *in vitro* to inhibit pre-adipocyte differentiation into adipocytes and to demonstrate anti-allergic activity (29). The advances in the evidence about these compounds have led to widespread production of extracts from PHs frequently used in pharmaceuticals in most global regions (18, 25, 26). In addition to the potential pharmacokinetics of PHs, extracts from PHs have also been observed to reduce total pathogen activity when used as a food additive, benefitting preservation when incorporated into foods (30). This may explain why foodborne pathogens, such as mycotoxins (e.g., aflatoxin), are markedly reduced in the shells even among peanut lots with detected aflatoxin (31).

### Edible use of peanut hulls

Peanut by-products, including PHs, are currently legally recognized foods in the United States. While their use as culinary flour is described, flour made from PHs (PHF) is not currently processed for human consumption anywhere in the world, presenting an opportunity for public health researchers, producers, and subsequently global ingredients manufacturers. Once demonstrated to be acceptable to consumers, PHF added to baking flour as an amendment to common baked goods (e.g., breads, cookies, cakes, etc.) could increase total quantity and availability of foodstuffs for human consumption, while reducing the cost to produce them, which offers an important strategy for addressing food access especially for at-risk regions many of which overlap with peanut growing regions. Amendment with PHF also offers the ability for common foods to enhance product formulation healthfulness, owing especially to the abundance of health-promoting nutrients such as the phytonutrients (e.g., luteolin and resveratrol) in high concentration, as well as by increasing the relative proportion of protein and fiber (1, 16, 17, 23, 30).

Increasing the concentration of dietary fiber in common foods would have multiple expected beneficial effects for cardiometabolic health including impacts on satiety, gastric emptying, and fecal energy and cholesterol loss which help to facilitate healthy weight management and improved blood lipid and glucose management (32–34). Consistent with the global literature, obesity and related downstream cardiometabolic conditions are linked to increased consumption of food with greater energy density and that are low in dietary fiber and protein (35). Small shifts in composition resulting in less energy contribution from carbohydrates and more from protein through the inclusion of PHFs also offers opportunities by converting common wheat-based foods like breads and crackers into readily accessible and convenient foods with complementary proteins (sometimes referred to as complete proteins) (36, 37). Inclusion of PHF in convenient food sources would also contribute amino acids known to support skeletal muscle hypertrophy including leucine and arginine (Table 1), and thus amendment with PHF could help to support healthy body composition beyond weight management, help promote developmental linear growth in children, and safeguard healthy aging in older adults who often struggle to maintain lean mass (36–38). In addition to the benefits of improved dietary fiber and protein in foods, regular dietary intake of foods high in polyphenols and flavonoids (like those in abundance in PHs) have been associated with improved health-span including fewer cardiovascular events, less neurodegenerative and metabolic disease, and preserved cognitive function (20, 26, 39).

**Table 1:** Comparing estimated global volumes of major nutrients in the hulls of peanuts, hazelnuts, and almonds.

	Peanut hulls	Hazelnut hulls	Almond hulls
Estimated crop produced, annually	46.40	0.87	1.70
Estimated hull weight available to be valorized, annually	10.20	0.44	0.85
Nutrients in hull volumes			
Calories (million kcal)	43,788,600	1,744,785	3,243,600
Fat	1.530	0.002	0.028
Carbohydrates	0.204	0.044	0.255
Fiber	6.100	0.370	0.128
Lignan	2.244	0.196	0.077
Hemicellulose	1.097	0.096	0.051
Protein	0.724	0.013	0.018
Asparagine	0.098	–	0.009
Glutamine	0.078	–	0.003
Arginine	0.022	–	0.001
Leucine	0.046	–	0.001
Total essential amino acids	0.210	–	0.009

All units are in million US tons unless otherwise specified. Values represent means of cited literature including systematic review. Specific amino acid composition is not well described in the literature specific to hazelnut shell. However, based on Frazzini et al. (47) concentrations of these may be higher than the kernel by weight, albeit less than the skin (9, 27, 47–52).

Peanuts are palatable foods and in high demand globally. PHF has a peanut flavor and thus is likely to be rated as desirable and acceptable, enabling its use as a means of fortifying food commonly consisting of refined starches, like white bread, with additional fiber and protein. While increasing the intake of fiber, and reducing proportions of refined starches is a known approach to improve the healthfulness of foods (40), sources of dietary fiber and protein widely available in high-income countries (HIC) are scarce to non-existent in LMIC and thus the practice is less common in global regions most at-risk. However, PHs are readily available worldwide making them a practical, low-cost option as a global public health strategy. PHF could thus serve as a familiar, natural, whole-food ingredient to lower the cost of food, increase the abundance of foodstuffs, and help address nutrition security while addressing the double burden of weight management and cardiometabolic diseases at elevated risk in many peanut-producing regions (18, 21, 23, 25–28).

### Uses of other nut hulls

The valorization of other nut hulls and nut by-products is well-established for coconut, hazelnut, almond, and walnut (18, 30, 41). Some of these, like coconut fiber flour, are readily available in supermarkets of HIC. When used for culinary purposes, these flour alternatives reduce net carbohydrates through the addition of dietary fiber, increase the proportion of calories from protein, and add beneficial bioactives similarly to how PHF could be utilized (18, 30, 41). Like PHs, hazelnut hull and almond hull are known to be directly edible to humans and some initial development of food products utilizing these ingredients exist. Notably, hazelnut hulls have been incorporated into commercially available snack foods (42) and almond hulls have been added to bread, with favorable enjoyment and palatability by raters (43), and early work has explored using almond hulls to produce mycoprotein for alternative protein foods (44). While not all nut hull products are used to make edible goods (11, 45), the growth in the market demand for hazelnut hull flour (for all uses, including culinary) is expected to rise from USD 2.43 billion in 2022 to USD 5.48 billion by 2031, for one example (46). Prior reports on the human food valorization of nut by-products demonstrates that human ingestion is likely safe, feasible, and is supported by significant economic interest (42, 43).

Global production volumes vary substantially between peanut, hazelnut, and almond resulting in vastly different waste volumes—peanuts are grown in considerably larger volumes resulting in considerably higher waste volumes. Additionally, the nutritional compositions between hulls also varies considerably, with a much larger absolute amount of protein, fiber, and nutrients in reclaimed from PHs compared to almond or hazelnut hulls (see Table 1).

## Processing and manufacturing requirements

Methods for nut hull processing is well described but effective yields, especially for delicate phenols, from upcycling is a known challenge. For nut hull processing, thorough maceration and not just simple grinding results in greater phenol extraction volumes, and with chemical and enzymatic methods increased contact time of macerated substrates is also beneficial, in large part due to the high concentration of fiber (53–55). Most experiments aiming to increase total yield and maximize nutrient reclamation from nut hull waste include multi-step processing methods where pretreatments employ thermochemical approaches such as heated alkaline liquid for lignan removal, or heated weak acid treatment for hemicellulose removal followed by the main processing through hydrolysis or other extraction methods (53, 56). Using pre-treatment processes that remove or fractionate lignan and hemicellulose increase the ability to extract higher yields of protein, especially at lower temperatures, and methods that operate at lower temperatures allow for greater retention of polyphenols while using less energy.

The opportunity for the culinary use of PHF has been described in the literature for over 45 years. The earliest paper (1979) explored utilization of PHs as an additive stabilizer for peanut butter, and shortly thereafter PHF was explored as a nutritional amendment to bread flour (16, 17, 57). Following this early exploration there was a large gap in exploration of the food uses of PHs, however substantial exploration into PHs as a source for biofuel, filtration, and building materials commenced in the first two decades of the 21st century (10–12) alongside nutraceutical development (18, 23, 30, 58). In 2007, a U.S. patent describing basic oxidation-based (hydrogen peroxide) and alkaline-based methods (deionized water) followed by simple drying and grinding, and limited toxin remediation for the processing of PHF was submitted and subsequently abandoned (59). Despite these early descriptions, and many recent advances in low-energy and high-efficiency processing technology, no active patents exist for producing PHF, or for processing PHs for other human edible uses such as the production of protein isolates or protein-polysaccharide concentrates (akin to pea protein), or common plant-based additives and ingredients like cellulose food additives like methylcellulose. Below, we outline key recommendations on how to safely process PHs for human food, describing both modern and traditional technological methods suitable for small on-farm operations and major industrial commercial processors, sensitive to 1) safety; 2) texture; 3) nutrient extraction and concentration; and 4) potential future uses.

### Safety

#### Aflatoxin

A major consideration for preparing and processing peanut foods is food safety. Peanuts and all peanut by-products require similar food safety considerations to grains and other culinary nuts (30, 60–64). The most commonly discussed foodborne pathogen for peanuts is aflatoxin, a prevalent soil-dwelling mycotoxin that is common among nuts, seeds, and grains, and of particular concern for peanut production because peanuts mature underground (31, 65). Aflatoxin is highly toxic and carcinogenic to humans, and unlike most foodborne pathogens is not readily killed through cooking or standard dry processing. Accordingly, most countries have regulations that require aflatoxins in food and in animal feed to be <20 μg/kg, and most outline routine testing of in-shell, peanut kernel, raw, and roasted products and preventive control measures from field-to-fork. In the U.S., as much as 24% of peanut crops may be discarded annually due to aflatoxin (66, 67).

Despite direct contact with the soil, aflatoxin concentration in the hull is typically far lower than in the peanut kernels themselves, owing in part to hull anti-microbial properties, and to the relatively lower moisture level which mycotoxins need to colonize. Indeed, early separation of the hull from the kernel, and manual rather than mechanical separation is also associated with reduced or absent aflatoxin detected in tested samples because it lowers overall moisture, allows for more accurate visual inspection, and reduces early-stage processing-related transfer (31). Overall, visual inspection, sorting, drying, dry storage, roasting, and blanching are the preferred methods for reducing aflatoxin in nuts and grains and are commonly employed in the treatment of peanuts, including hulls (64).

#### Salmonella

Another major pathogen of concern for peanuts is salmonella (68). Unlike aflatoxin, salmonella survives well in dried foodstuffs like hulls and flour but is readily killed through standard cooking and processing, including baking and roasting. The primary source of salmonella in peanuts comes from nearby cultivation of chicken livestock, which can deposit salmonella-infected feces on fields, or contaminate the water used to irrigate peanut crops (68).

#### Prevention, remediation, and best practices

The best method to prevent aflatoxin and salmonella infection in peanut crops is routine testing of soil, water, and raw products as well as the frequent sanitization of harvesting and processing machinery. In the case of aflatoxin, additional field methods to reduce infection include the use of resistant strains (e.g., FloRun^™^‘107’ and Tifguard^™^20), calcium and lime soil amendments, proper irrigation, pest control, earlier harvest (69, 70), thorough drying before combined processing (67, 71), manual selection and sorting (31, 61, 70), early separation of hulls for dry processing, and representative testing requirements which are already the norm in peanut production protocols (70). For salmonella, guidance is to restrict possible chicken farm contamination on land and prevent it from entering the water supply, and then to use heat processing for foods; however, additional elimination of salmonella can be accomplished through storage at ambient temperatures above 82 degrees Fahrenheit with durations over 270 days (68). Preventing contamination and cross-contamination from equipment can also limit pathogen activity, and some processing methods can remove pathogens allowing exposed yields to return to human consumption (with duly required documentation) (31, 72). Safety measures would precede grinding PHs into flour for baking or undergoing further processing like protein or dietary fiber extraction. (For an overview of processing steps, see Table 2 for preparing peanut hull for flour and Table 3 for preparing peanut hull for extraction).

**Table 2:** Preparation steps to produce peanut hull flour for baking.

Processing for flour			
	Chemical or bioactive reagent	Damage control	Moisture reduction
Field methods to reduce pathogen activity	Lime and calcium soil amendment; *A. flavus* or other resistant strain competition		
Harvest methods to reduce pathogen activity		Manual Inspection	Rapid Shelling
Systematic sampling		Pathogen testing to initiate effective quarantining and discarding, as well as soil amelioration	Moisture testing to ensure low water activity inhospitable to microbial growth
Wet sanitization	Hydrogen peroxide, hypochlorite, electrolyzed water, and others may kill and in some cases remove mycotoxins		
Texture maturing and conditioning	UV irradiation to mature flavor and enhance milling texture; sodium bicarbonate and sodium bisulfate to reduce mixtures and produce finer flour		
Rapid drying			Baking, roasting, fan, and sun drying. UV irradiation for rapid drying, producing finer and more consistent milling textures while controlling toxigenic species; Ozone gas may also be used, but is best applied after milling to support finer textures
Milling and equipment sanitization	Burr grinders should be sanitized with food-safe dilutions of hypochlorite, or equivalent, in between each processing lot and at the end of working periods to reduce equipment-related pathogen transfer		
Bagging and storage			Fully dried, milled flour should be tested for water activity, and stored as flour in rooms without the threat of flood or high humidity

**Table 3:** Preparation steps to produce peanut hull flour for nutrient extraction.

Processing for nutrient extraction			
	Chemical or bioactive reagent	Damage control	Moisture reduction
Field methods to reduce pathogen activity	Lime and calcium soil amendment; *A. flavus* or other resistant strain competition		
Harvest methods to reduce pathogen activity		Manual Inspection	Rapid Shelling
Systematic sampling		Pathogen testing to initiate effective quarantining and discarding, as well as soil amelioration	Moisture testing to ensure low water activity inhospitable to microbial growth
Texture maturing and conditioning	UV irradiation to mature flavor and enhance milling texture; sodium bicarbonate and sodium bisulfate to reduce mixtures and produce finer flour		UV irradiation also assists drying, producing finer and more consistent milling textures while controlling toxigenic species; Ozone gas may also be used but is best applied after milling to support finer textures which may improve protein yield
Milling and equipment sanitization	Burr grinders should be sanitized with food-safe dilutions of hypochlorite, or equivalent, in between each processing lot and at the end of working periods to reduce equipment-related pathogen transfer		
Preparation for extraction (by method)	Dissolved in water or other solvents, specific to each method. Additional use of food-safe acids and enzymes may also be applied to extract one or more nutritional compounds		Dry methods may include ultrasound- and microwave-assisted extraction, or high-pressure extractions without direct water contact, enabling less need for drying final substrates
Nutrient retention and single-step production of multiple ingredients	Different heat, enzymes and multiple pass methods may be used to obtain the maximum recycled yield from materials with single processing		
Bagging and storage			Fully dried, powdered extracts should be tested for water activity, and stored in sealed, air-tight containers impervious to moisture infiltration

Methods to salvage peanut crops are important given the high percentage of loss. In the case of aflatoxin, the persistence of the mycotoxin in soil can negatively impact farmer livelihood for years, and increase the risk of exposure for local and global populations (70). Extensive industrial chemical methods to control mycotoxins are well-described (70, 72–75). Additionally, some simple and readily available methods exist that are accessible to small-scale and on-farm processors, are compatible with organic farming, and are recognized as acceptable to consumers: 1) five-minute exposure to medium power in a microwave oven has been shown to reduce aflatoxin in peanut kernels by as much as 50%; 2) sodium bicarbonate (baking soda) baths have been demonstrated to reduce aflatoxin by as much as 51%; and 3) common household sanitizing solutions such as sodium hypochlorite (bleach) diluted in water at concentrations of 1–2% effectively eliminate aflatoxin in food and on equipment; bleach concentrations of ≤3% are considered safe and are commonly used to sanitize foods and food equipment, but should be followed by a solute rinse (76).

In addition to the methods described above, the use of atoxigenic strains of aflatoxin (e.g., isolates of *A. flavus*) is a form of biocontrol increasingly used to mitigate toxigenic aflatoxin in foods (73, 74, 77). These biocontrol agents can be applied in the field to prevent infection, spread, and control outbreaks in previously exposed soil, and are an inexpensive means of safeguarding harvests, livelihoods, and bolstering nutrition security.

#### Allergenicity

While the allergenicity of nuts is often discussed, peanut allergies impact <2% of the global West, and with significantly lower prevalence of allergies in Africa, Asia/Oceania, and Central/South America where lifelong immunity is entrained by routine peanut consumption during pregnancy and lactation (78). Additionally, nut hulls are typically less allergenic than nut kernels and some processing may further decrease allergenicity (79).

### Texture

Thorough drying, oxidizing, and low-tech processing methods such as freezing and multi-pass burr grinding as well as modern sustainable methods like ultrasonic pulsation can help achieve fine textures for milled PHF. Advanced methods like ultrasonic pulsation (also known as ultrasound-assisted extraction, or UAE), can also assist with microbial control and nutrient recapture in extraction (1, 27, 80–82). In particular, combinations of weak acids (e.g., citric acid), electrolyzed water (where electricity is passed through an electrolyte-rich fluid), and reducing agents (e.g., sodium bisulfate and sodium bicarbonate) at various stages in wet processing can serve dual functions for safety and texture conditioning suitable for small, on-farm operations (83). Additionally, ultraviolet (UV) irradiation, a common sanitization method in the food industry, is a very effective maturing agent for grain and seed which can be employed with PHs to develop flavor, accelerate drying, and reduce milled particle size to achieve finer textures (84). On the other hand, ozonation (exposure of plant materials to ozone gas) has been shown to increase particle size when seeds are exposed before milling; in order to enhance texture, use of ozone sanitation may be best for milled flour rather than as a pre-treatment for whole PHs.

### Extraction

Different extraction methods influence the yield for recapturing the target nutrient (e.g., protein, dietary fiber) and the retention of other nutrients (e.g., polyphenols) (27, 80, 82, 85). Methods like UAE and other recent advanced methods of sustainable processing such as critical fluid-assisted extraction are able to process plant food materials at lower temperatures and without caustic solvents resulting in recapture of some of the highest yields for nutrients like protein. Enzymatic-assisted extraction uses less power than many other methods and uses milder and often natural chemical processing to break down complex plant cell structures while retaining protein-polysaccharide compounds in the finished product. Microwave-assisted extraction is able to quickly and efficiently break down complex food matrices offering time advantages for producers that can translate to increased yield per energy used when scaling and thus greater efficiency of throughput. Pulsed-electric-field-assisted extraction is a targeted electric extraction method that is very time and energy efficient and reduces the need for energy use in drying steps common in wet solvent-based extractions and have also received recent attention due to the improvements in food safety, nutrient yield, reduced energy and other natural resource demands, and retention of nutrients compared to standard methods. These sustainable processing methods have also been shown to suppress microbial and pathogen activity, to produce superior protein extraction yields often between 86 and 95%, to retain antioxidant polyphenols, and may even be able to concentrate flavorful amino acids (e.g., aspartate and glutamate) making them especially attractive for use in plant-protein production (86). Processing PHs to extract protein can create complex protein-polysaccharide concentrates, akin to other legume protein concentrates like pea protein, and efficient modern methods can use single-step processing to produce both protein isolates and functional cellulose ingredients for other consumable purposes (e.g., dietary fiber supplementation, or as emulsifiers and thickeners used in processed foods)—all while being more energy and water efficient than previously needed (82). These processing methods together with advancements in agricultural methods form the so-called “green revolution” enabling a food system that is more efficient at producing high food volumes and that can more efficiently reclaim food waste and by-products (27, 48, 49, 80, 85, 87).

### Other culinary uses

#### High-fiber uses

The proposed use of PHF as a baking flour amendment in breads, crackers, and biscuits has been previously described (17, 59). Prior studies evaluated the physiochemical enhancements and consumer acceptance of almond hull added as an amendment to baking flour in bread (43) and hazelnut flour in snack foods (42). These additions demonstrated not only an increase in the available polyphenols of those foods, but addition of these ingredients also resulted in considerable improved textural conditioning, shelf life, and higher taster rating compared to comparator foods made with traditional recipes. Fiber-rich hull flours can enhance baking textures thanks to their strong binding capacity and higher water absorption (1). Owing to the large concentrations of cellulose, hemicellulose, pectin, and lignan in proportions similar to other plant materials used to capture fiber for supplements and food processing ingredients, PHs offer a particularly rich and underutilized source for these resources (1).

Through the addition of PHF, common foods (both packaged and cooked at home) such as baked goods like bread and cookies, as well as stews and gravies can benefit from the inclusion of PHF. Such inclusion can serve to lower the total calories consumed (38). Furthermore, because dietary fiber acts as a binding agent to bile acids and dietary fats increased intakes in common foods could have important impacts on circulating cholesterol and dietary fat absorption, while also serving to feed beneficial gut microflora given the role of dietary fiber as a prebiotic (1).

Aside from direct culinary applications to improve the nutritional and functional properties of flour, PHF can be hydrolyzed to extract concentrations of lignans for nutraceutical use, leaving the remaining cellulose for applications such as methylation into methylcellulose (a common fiber supplement, and a surfactant used in the food industry as an emulsifier, thickener, and binding agent known as e461) (88). Methylcellulose is used in a range of food products, from baked goods like bread and cakes, to dairy products like condensed milk and ice cream, as well as plant-based alternative foods. However, targeted processing or activation of protein during the extraction could enable more nutritious protein-polysaccharide blends that retain useful rheological properties associated with cellulose additives, resulting in greater utilization of the total volume of wasted PHs improving the cost effectiveness of upcycling, providing greater culinary versatility, an enhanced nutritional profile, with fewer overall processing demands. Through various titrations of fiber content, such as through enzymatic or oxidative pre-treatment processing with varied contact time, or the extraction methods mentioned above, PHs can be manipulated to create nutrient-rich and natural peanut-based cellulose emulsifiers similar to one of its earliest described uses—added back into peanut butter as a stabilizer and prevent separation (16). In the case of adding PHs to peanut butter, the consumer acceptance was notably improved due to the textural enhancements and was not impacted by the use of a food additive because peanut ingredients are overall rated highly and of course expected in peanut butter.

#### Protein recapture

The amino acid composition of PHs has a relatively high concentration of branch-chain amino acids which are associated with muscle protein synthesis (36, 37, 89) (Table 1) and thus PHs may be favorable for athletic uses, in addition to their high concentration of flavorful amino acids which may make them especially desirable in the culinary development of alternative and plant-based protein foods. Arginine, leucine, asparagine, and glutamine are among the most abundant amino acids in PHs (Table 1), associated with muscle synthesis and neuro-protective benefits, and are also associated with umami flavors in foods and especially with grilled and aged meats (20). Thus, the inclusion of these upcycled plant foods in alternative protein foods may help to enhance not only the texture but the flavor of finished products while utilizing ingredients sourced from preferred and familiar food crops.

The protein content of PHs have many possible applications, including as isolates with or without large fractions of dietary fiber. For example, PHs can be processed like other popular plant-based proteins, (e.g., pea protein) which are typically complex polysaccharide-protein concentrates with approximately 70–77% protein, with most of the remaining components fiber and digestible carbohydrates. Healthy plant proteins like pea protein have been used in multiple functional applications, including as protein supplements and in the development of plant-based and cell-cultivated animal foods. At the current moment, pea protein is a premium product, with large volumes of commodity legumes grown specifically to meet the demand, and estimated demand is soon expected to outsize production capability (90). Recapturing lost protein from an alternative legume source like PHs could provide an additional source to meet that demand while increasing the efficiency of existing production systems. A conservative estimate of the yield possible from PHs would produce 595,000 tons of protein alone (1, 82), a production volume comparable to the market demand of two small countries with public data available on the tonnage of pea protein used (Saudi Arabia and United Arab Emirates) (91). Given the likelihood of creating protein-polysaccharide compounds similar to pea protein, the final sale volumes would be even larger (approximately 20% larger if aiming for protein concentrations similar to pea protein) due to retained fiber and carbohydrate content. The protein concentrates derived from PHs could be used as a mildly peanut-flavored protein supplement for use in sports drinks and powders; as part of the protein base for plant-based alternative meat and dairy; as a protein and fiber rich component of cookies and other baked goods; and potentially as a replacement for other plant proteins used in the serum growth media, bioink, and structural scaffolding components of cultivated meat and novel animal food alternatives (92).

## Conclusion

In this review, we have outlined several methods that could be used to convert peanut hulls (PHs) into safe, high-value commercial products for human consumption. The combination of the above methods along with standard Good Manufacturing Practices (GMPs) could be implemented safely and inexpensively using materials available to farmers and processors of all sizes. As the global population increases, so does the prevalence of cardiometabolic disease, necessitating innovative strategies to provide health-promoting food to a record number of people. One important strategy to address increased food production needs and the availability of healthy foodstuffs is to optimize food supply streams by increasing the efficiency of agricultural outputs. More efficient systems enable the retention of more plant foods grown and can reclaim nutrients for human consumption that are lost by treating these food byproducts as waste. Resource efficiency thereby can reduce the impact of food systems on the planet by drawing less resources per ton produced, and by creating fewer greenhouse gases emitted from food waste—itself a major contributor of emissions (7, 85, 93). Reducing food waste and improving food system efficiency through valorizing edible food byproducts for human consumption also may create additional revenue streams for peanut farmers who currently divert over 22% of their crop to low-value streams like animal feed and who are typically the most disadvantaged agents in food systems and often experience nutrition insecurity themselves.

Edible PHF has been explored for over 45 years, but the human food use is virtually non-existent today. Typically, only nutraceutical products such as phenol extracts are produced commercially. Nonetheless, our review shows that PHs could be processed safely for use as a readily available foodstuff that may offer substantive benefits for multiple stakeholders in the food supply chain, especially for the >99% of the global population who can safely consume it. Following the steps we outline, safe valorization of PHs could be routinely processed at grower sites, in processing facilities, and safely incorporated into prepared foods already widely consumed in the human food supply. When processed into flour, PHF can be used to rebalance the nutritional composition of common foods like bread, which is an underutilized approach to help address the obesity pandemic and potentially aid ongoing efforts to reduce the global burden of cardiometabolic disease. Additionally, a large amount of healthful and pleasant-tasting plant protein can be recaptured from PHs to increase the total global protein supply and support ongoing efforts to improve the functionality and affordability of alternative proteins.

With more coordinated efforts to valorize PHs, the current processing efforts that capture some of the beneficial polyphenols in PHs could enable streamlined and cost-effective processing of PHs for direct human food consumption. Overall, developing human food uses for PHs is consistent with priorities to transition to a more efficient food system that is more sustainable for the people who grow the food, the planet it grows on, and the people who eat the food.

## Limitations and future research

Valorizing edible food waste for human consumption increases the amount of sellable yield farmers can monetize. While other studies indicate that the transfer of plant byproducts from animal feed and industrial building materials offers considerable economic advantages to farmers and other stakeholders in the supply chain, this mini review was not intended to be a comprehensive economic analysis. Future research should evaluate the revenue opportunities and cost savings to farmers and distributors, and to evaluate whether other farming sectors experience any secondary impacts by diverting PHs for human consumption. Additionally, a more thorough life-cycle analysis would be needed to quantify the cradle-to-grave environmental impacts of upcycling PHs, as well as to evaluate the tradeoffs among various uses of PHs. Different technological methods described in the above sections demand differing levels of water, power, and other inputs and produce different processing byproducts which may differ compared to simple on-farm processing of PHs. Future research using techno-economic analyses sensitive to full life-cycle assessments should precede full scale commercialization of PHF.

During our review, we discovered that quantification of micronutrients and specific amino acids for some nut byproducts, such as hazelnut shells, is underrepresented in the literature. The literature quantifying nutrient composition of nut by products also indicates great variation in available nutrients likely owing to growing conditions, climate, processing, and storage among other factors. Future research may wish to identify which nut byproducts are ideal for human food recapture based on nutrient availability, compared to other upcycling efforts such as biofuel.
